# Transforming mental health: the future of personalized psychobiotics in anxiety and depression therapy

**DOI:** 10.3389/fnins.2025.1711846

**Published:** 2025-11-26

**Authors:** Gilberto Uriel Rosas-Sánchez, José Luis Muñoz-Carrillo, Cesar Soria-Fregozo

**Affiliations:** 1 Programa de Estancias Posdoctorales por México, Centro Universitario de los Lagos, Universidad de Guadalajara, Lagos de Moreno, Jalisco, Mexico; 2 Departamento de Ciencias de la Tierra y de la Vida, Centro Universitario de Los Lagos, Universidad de Guadalajara, Lagos de Moreno, Jalisco, Mexico

**Keywords:** anxiety, depression, gut-brain axis, probiotics, psychobiotics

## 1 Introduction

The incidence and prevalence of anxiety and depression continue to rise, and conventional pharmacotherapies offer only incomplete solutions for millions of patients around the world. At the same time, scientific insights into the microbiota-gut-brain axis have opened up unprecedented opportunities for therapeutic innovation. Recent literature suggests a positive role for probiotics in the treatment of depression and anxiety (Merkouris et al., 2024); however, the heterogeneity of therapeutic responses suggests that current approaches do not account for individual differences in microbial ecology and host-microbe interactions (Alberdi et al., 2022). The concept of psychobiotics—probiotics with positive effects on mental health—has evolved from empirical observations to a mechanistic understanding (Sarkar et al., 2016; Sharma and Bajwa, 2021). However, the field is at a critical juncture where generic interventions must give way to personalized approaches (Erikainen and Chan, 2019). The rapidly growing field of microbiome-directed therapeutics has attracted significant attention due to its potential to revolutionize healthcare (Yaqub et al., 2025) and represents a fundamental shift from population-based to precision medicine paradigms. This transition is particularly urgent as understanding how broad host-microbiome associations are maintained across populations reveals individualized host-microbiome phenotypes that can be integrated with other “omics” datasets to improve precision medicine (Bashiardes et al., 2018; Lloyd-Price et al., 2019). This opinion piece will address the potential of probiotics and psychobiotics as precision medicine tools in the treatment of anxiety disorders and depression. The current state of scientific evidence, the neurobiological mechanisms involved, limitations of the field, and future prospects for the design of individualized interventions will be discussed, with an emphasis on the integration of “omics” approaches and bioinformatics technologies.

## 2 Mechanisms beyond traditional neurotransmission

### 2.1 Neurotransmitter production and modulation

**Figure 1:**
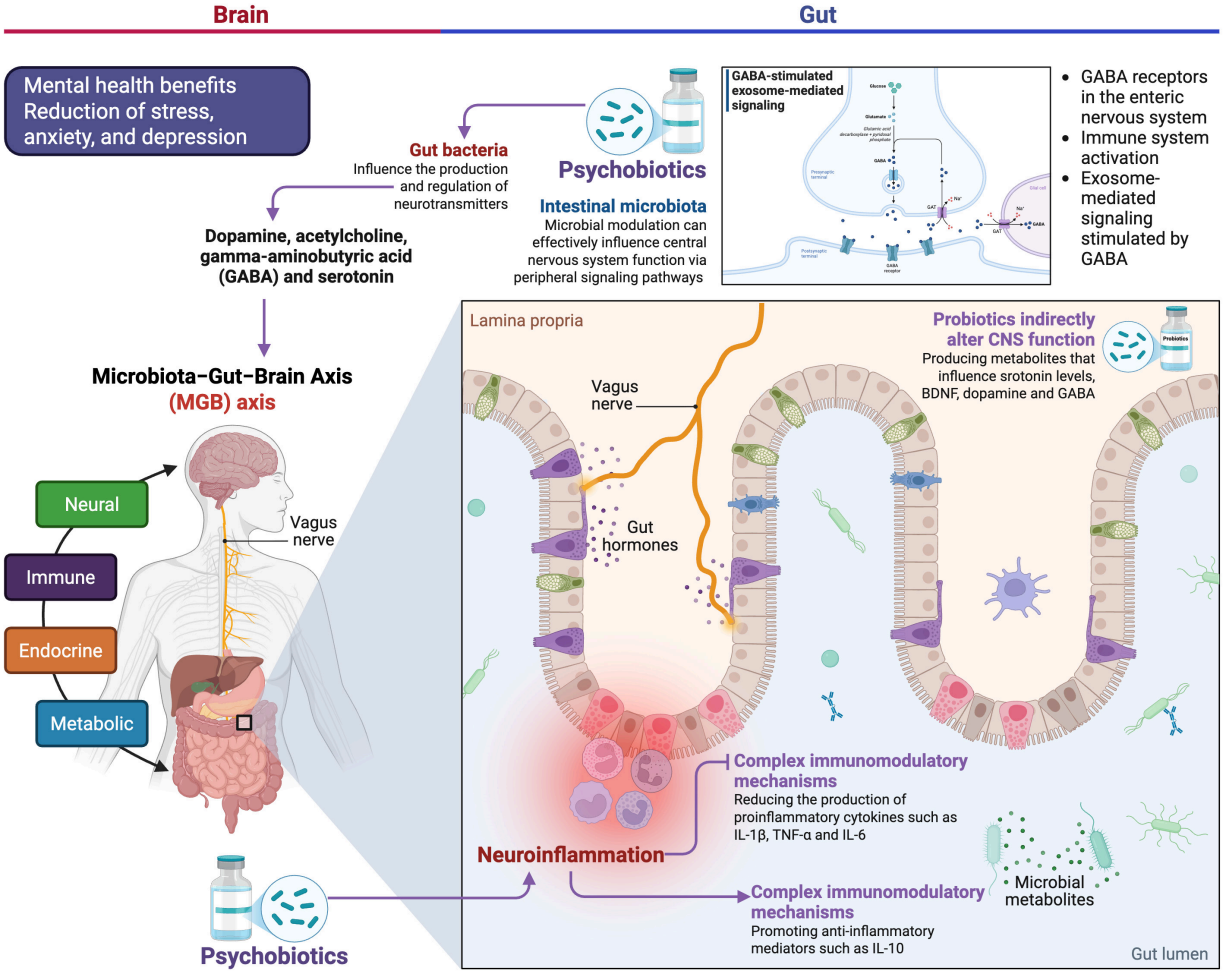
Neurochemical and anti-inflammatory mechanisms of psychobiotics through the gut-brain axis. Psychobiotics act beyond traditional psychiatric pharmacology by modulating neurotransmitter production (dopamine, acetylcholine, GABA, serotonin) and regulating immunological and inflammatory processes through the gut-brain axis. These mechanisms include the direct synthesis of neuroactive compounds, the activation of GABA signaling networks in the enteric and immune nervous systems, and the reduction of proinflammatory cytokines (IL-1β, TNF-α, and IL-6) along with the increase of anti-inflammatory mediators (IL-10), generating a comprehensive and synergistic therapeutic effect. Figure created with BioRender.com.

Research demonstrates substantial evidence for probiotic production and modulation of neurotransmitters through the gut-brain axis, the mechanistic basis for the efficacy of psychobiotics goes far beyond traditional psychiatric pharmacology (Rosas-Sánchez et al., 2025). Gut bacteria can influence the production and regulation of neurotransmitters such as dopamine, acetylcholine, gamma-aminobutyric acid (GABA) and serotonin and affect their availability in the brain (Rosas-Sánchez et al., 2025; Kyei-Baffour et al., 2025). Certain bacterial strains show a remarkable ability to directly synthesize neurotransmitters, with several species being among the documented producers of neuroactive compounds (Oroojzadeh et al., 2022). Recent discoveries in neuromicrobiology have elucidated sophisticated signaling networks (Jameson et al., 2020). GABA signaling networks in the brain-gut-microbiome axis involve multiple pathways, including activation of GABA receptors in the gut nervous system, activation of the immune system, and GABA-stimulated exosome-mediated signaling (Belelli et al., 2025). This multi-target approach distinguishes the psychobiotic mechanisms from conventional antidepressants, which generally modulate single neurotransmitter systems. The clinical relevance of these mechanisms is demonstrated by direct interventions showing that targeted microbial modulation can effectively influence central nervous system function via peripheral signaling pathways (Cocean and Vodnar, 2024), providing tangible evidence for the therapeutic potential of precision microbiome interventions (Figure 1).

### 2.2 Neuroinflammatory pathways and systemic effects

In addition to the production of neurotransmitters, psychobiotics influence neuroinflammation through complex immunomodulatory mechanisms reducing the production of proinflammatory cytokines such as IL-1β, TNF-α, and IL-6, while promoting anti-inflammatory mediators such as IL-10 (Rosas-Sánchez et al., 2025; Oleskin and Shenderov, 2019). The gut-brain axis is a bidirectional communication network that connects the enteric and the central nervous system (CNS) and also includes endocrine, humoral, metabolic and immunological communication pathways (Carabotti et al., 2015). This comprehensive network enables the simultaneous influence of inflammation, neurotransmission and neuroendocrine function. Probiotics indirectly alter CNS function by producing metabolites that influence serotonin levels, brain-derived neurotrophic factor (BDNF), dopamine and GABA (Dziedzic et al., 2024; Kimse et al., 2024). The integration of these mechanisms leads to synergistic therapeutic effects that go beyond individual signaling pathways and create a comprehensive neurochemical network suitable for microbial interventions (Figure 1).

## 3 Meta-analytical insights from recent literature

The clinical evidence for the efficacy of psychobiotics has evolved considerably, with recent meta-analysis providing nuanced perspectives on therapeutic potential (Vasiliu, 2023; Mosquera et al., 2024). A random-effects meta-analysis of 34 controlled clinical trials investigating the effects of prebiotics and probiotics on depression and anxiety found heterogeneous but promising results (Liu et al., 2019; Zandifar et al., 2025). In particular, taking probiotics for up to 4, 8, and 12 weeks is effective in reducing depressive and anxiety symptoms in clinically diagnosed patients compared to placebo (Lew et al., 2019; Asad et al., 2025). However, the evidence also has important limitations. It may be premature to conclude clinical efficacy in relieving anxiety, as the effect size is small and there is no consensus on the optimal dose, treatment duration or specific formulations (Zhao et al., 2023). These findings highlight the need for a personalized approach that takes into account individual variability in response to treatment. Recent analyses indicate that specific strains—such as *Lacticaseibacillus casei* Shirota, *Lactobacillus gasseri* CP2305, *Lactiplantibacillus plantarum* PS128, *Lacticaseibacillus rhamnosus* LR06, and *Bifidobacterium longum* subsp. *longum* R0175 have shown efficacy in alleviating anxiety and depression symptoms (Ross, 2023; Rahmannia et al., 2024). This strain-level activity supports the development of precision psychobiotic interventions.

### 3.1 Individual variability and response prediction

The heterogeneity in treatment responses across studies reflects fundamental individual differences in microbiome composition, host genetics, and environmental factors (Schupack et al., 2022). The current review suggests that probiotics may improve symptoms of depression and anxiety in clinical patients; however, given the limitations in the included studies, randomized controlled trials (RCTs) with long-term follow-up and large sample sizes are needed (Minayo et al., 2021). This variability represents both a challenge and an opportunity for precision medicine approaches. Understanding the biological basis for differential responses will enable stratification of patients based on predictive biomarkers (Yang et al., 2024), optimizing therapeutic outcomes while minimizing unnecessary interventions. It is essential to recognize that, despite advances, the heterogeneity in clinical study results reflects the complexity of the microbiome and its interaction with genetic and environmental factors. The lack of standardized protocols, in addition to variability in individual microbial composition, limits the reproducibility and clinical applicability of probiotic interventions. These aspects should be considered in future research to optimize their clinical utility.

## 4 Individual microbiome signatures and response prediction

The transition to precision psychobiotics requires a fundamental reconceptualization of therapeutic approaches based on individual biological profiles (Slykerman et al., 2025). The concept of the “precision microbiome” involves the precise analysis and typing of the microbiota in specific hosts using advanced tools such as high-throughput sequencing, genomics and artificial intelligence. This technological foundation enables comprehensive characterization of individual microbiome signatures that could predict treatment response. The inherent individuality of the composition of the microbiome requires personalized therapeutic strategies rather than one-size-fits-all solutions (Fang et al., 2025). The genomic revolution promises to transform our approach to treating patients by individualizing treatments, reducing side effects and lowering healthcare costs (Huang et al., 2023), with microbiome analysis being a critical component of this transformation.

### 4.1 Technological integration and therapeutic optimization

Advanced analytical approaches are revolutionizing the personalization of treatments by integrating multiple data streams (D'Urso and Broccolo, 2024). Precise reconstitution of the microbiome using high-throughput sequencing and artificial intelligence tools enables real-time monitoring and optimization of therapeutic interventions based on the dynamics of the individual microbiome (Fang et al., 2025; Huo and Wang, 2024). The application of these technologies facilitates unprecedented precision in the selection and monitoring of therapies. A new approach to treating disease through personalized probiotic therapies—also known as precision medicine—has been proposed by researchers (D'Urso and Broccolo, 2024; Huo and Wang, 2024), demonstrating the feasibility of individualized microbial interventions in various therapeutic areas. The integration of ‘omics' technologies—such as genomics, metabolomics, and microbiomics—allows for the identification of specific microbial profiles associated with positive or adverse responses to probiotics. These strategies pave the way for personalized therapies, where the selection of specific strains and combinations is tailored to a patient's unique microbial signature. Recent studies have exemplified how the analysis of these signatures can predict treatment efficacy, reaffirming the importance of a personalized medicine approach.

## 5 Future perspectives: toward psychobiotic medicine

The future of precision psychobiotics requires a deeper mechanistic understanding of host-microbe interactions in mental health (Slykerman et al., 2025). Psychobiotics are probiotic strains capable of influencing the gut-brain axis and have been shown to be effective in several neurological disorders (Oroojzadeh et al., 2022). To make progress in this field, a comprehensive mapping of microbial metabolic pathways, host genetic variants and environmental factors influencing therapeutic outcomes is required (Kwok et al., 2021). In psychobiotics, the potential of probiotics to influence the nervous system and mental health is being investigated through a comprehensive analysis of their effects on mood, cognition and stress response (Cocean and Vodnar, 2024). This mechanistic foundation will enable the rational design of personalized treatment regimens optimized for individual patients. The transition from research to clinical practice requires addressing multiple challenges while taking advantage of the opportunities that arise. Standardization of analytical protocols, validation of predictive biomarkers and integration into existing psychiatric care are crucial prerequisites for implementation (Kyei-Baffour et al., 2025; Liu et al., 2025). For example, in a recent pilot study, patients with major depressive disorder showed significant improvements after receiving a personalized probiotic regimen based on their baseline microbiome profile (Kreuzer et al., 2022; Gawlik-Kotelnicka et al., 2023; Johnson et al., 2025). These experiences, although preliminary, illustrate the feasibility and potential of personalized microbiome-centered therapies and underscore the need for larger-scale controlled trials. The development of precision psychobiotics requires interdisciplinary collaboration between microbiologists, psychiatrists, bioinformaticians and regulatory specialists. This collaborative approach will accelerate the translation of mechanistic insights into clinically applicable interventions to improve patient outcomes. The development of personalized microbiome therapies faces significant challenges, including regulatory hurdles, the costs of omics analysis, and ethical issues related to microbiome modification. Furthermore, it is essential to define reliable predictive biomarkers and establish clear clinical criteria for their use. Multidisciplinary collaboration, integrating microbiology, psychiatry, bioinformatics, and regulation, will be key to overcoming these barriers and bringing these innovations into routine clinical practice.

## 6 Discussion

The emergence of precision psychobiotics represents a convergence of multiple scientific advances that collectively challenge traditional approaches to mental health treatment. The evidence synthesized in this review reveals both unprecedented opportunities and significant challenges that must be addressed to realize the full therapeutic potential of personalized microbiome interventions. The mechanistic foundations for psychobiotic efficacy are increasingly well-established, with robust evidence for neurotransmitter production (Sarkar et al., 2016; Belelli et al., 2025), neuroinflammatory modulation (Carabotti et al., 2015; Kimse et al., 2024), and multi-pathway signaling networks (Cocean and Vodnar, 2024). However, translating these mechanistic insights into clinically effective interventions remains complex. The heterogeneity observed in meta-analysis (Liu et al., 2019; Zandifar et al., 2025; Zhao et al., 2023) suggests that individual differences in microbiome composition, host genetics, and environmental factors significantly influence treatment outcomes. This variability underscores the necessity for precision medicine approaches that can stratify patients based on predictive biomarkers (Yang et al., 2024; Fang et al., 2025). The challenge lies in identifying which combinations of microbial, genetic, and clinical factors best predict treatment response, requiring sophisticated analytical approaches that integrate multi-omics data (Bashiardes et al., 2018; Lloyd-Price et al., 2019).

Several critical limitations constrain current progress toward precision psychobiotics. The complexity of microbiome-brain interactions creates analytical challenges that exceed current technological capabilities (Lloyd-Price et al., 2019; Turnbaugh et al., 2007). Additionally, the temporal dynamics of microbial communities introduce variability that complicates therapeutic monitoring and optimization. RTCs have shown that probiotic treatment for 4 and 12 weeks does not pose a relative risk of treatment-associated adverse events, even in patients with irritable bowel syndrome (Mosquera et al., 2024; Hempel et al., 2011). The preponderance of evidence, including the long history of safe use of probiotics, as well as data from clinical trials and animal and *in vitro* studies, supports the assumption that probiotics are generally safe for most populations (Pinto-Sanchez et al., 2017). Future research should address heterogeneity in diagnosis and intervention types to better understand their efficacy (Moshfeghinia et al., 2025). The field requires rigorous adherence to evidence-based standards while resisting pressure for rapid commercialization of insufficiently validated interventions. Long-term safety considerations remain largely unexplored, particularly regarding potential unintended consequences of targeted microbial manipulation (Merkouris et al., 2024). Comprehensive safety assessment protocols must accompany therapeutic development to ensure patient welfare throughout treatment courses.

The transition to precision psychobiotics will require fundamental changes in clinical practice patterns, including integration of microbiome analysis into psychiatric assessment protocols (D'Urso and Broccolo, 2024; Kreuzer et al., 2022). This transformation necessitates substantial investment in diagnostic infrastructure, clinician training, and regulatory frameworks that can accommodate personalized interventions while maintaining safety standards. The economic implications are equally significant, as precision medicine approaches typically involve higher upfront costs for diagnostic testing and personalized formulation development. However, the potential for improved treatment outcomes and reduced healthcare utilization costs may justify these investments over the long term.

The evolution from population-based to precision-based psychobiotic interventions represents a paradigmatic shift in mental health therapeutics. Recent studies investigating the effects of probiotics on depression, anxiety and psychological stress provide the foundation for this transformation (Reis et al., 2020), but realizing the full therapeutic potential requires embracing personalized approaches that account for individual microbial signatures and host-microbe interactions. The convergence of advanced analytical technologies, mechanistic understanding, and clinical need creates unprecedented opportunities for therapeutic innovation. Success requires sustained commitment to rigorous scientific standards, collaborative research approaches, and patient-centered care models that prioritize safety and efficacy over commercial expediency. The future of mental health therapeutics lies not in generic interventions but in sophisticated, personalized approaches that leverage individual biological signatures to optimize therapeutic outcomes. Precision psychobiotics represent the next evolutionary step in this journey, promising more effective, safer, and more individualized treatments for anxiety and depression.

As we advance toward this future, the mental health community must embrace the complexity inherent in personalized medicine while maintaining focus on the ultimate goal: improving patient outcomes through scientifically rigorous, individually tailored therapeutic interventions. The transformation of probiotics from generic supplements to precision medicine tools represents both a scientific achievement and a moral imperative to provide the most effective possible care for patients suffering from anxiety and depression. This article proposes an innovative approach that combines recent scientific evidence, critical analysis, and a forward-looking perspective that emphasizes the convergence of microbiology, neuroscience, and personalized medicine. Its originality lies in contextualizing probiotic therapy within the precision medicine paradigm, offering both conceptual and practical insights, and proposing concrete pathways for its integration into clinical care.
